# Feelings and Experiences of Return‐to‐Work Among Nurses Occupationally Infected With COVID‐19 During the Late Phase of the Pandemic in Japan

**DOI:** 10.1111/nhs.70307

**Published:** 2026-02-17

**Authors:** Noriko Shinkai, Kayoko Ohnishi, Hisako Yano

**Affiliations:** ^1^ Department of Nursing, Faculty of Health Sciences Aomori University of Health and Welfare Aomori Japan; ^2^ Graduate School of Nursing Nagoya City University Nagoya Japan; ^3^ Graduate School of Nursing Aichi Medical University Nagakute Japan

**Keywords:** COVID‐19, feelings, interview, late phase of the pandemic, nurses, occupational infection, qualitative study

## Abstract

This study explored the feelings of nurses occupationally infected with COVID‐19 during the late pandemic phase in Japan, from diagnosis to return to work, to provide suggestions for safe and continuous employment. Semi‐structured interviews were conducted among 11 nurses infected during the late phase of the pandemic (August 2022 to February 2023). The data were analyzed using a qualitative inductive approach. At diagnosis, nurses experienced shock and a strong sense of guilt and self‐blame arising from the feeling of being blamed by others. Negative emotions during the infection period influenced postreturn feelings, leading to distrust stemming from insufficient infection control measures and a desire to leave their workplace. Conversely, gratitude for the support from managers and colleagues during the recuperation period fostered their determination to return. Furthermore, a sense of achievement, such as improved nursing expertise and reaffirmation of professional values, facilitated a positive transformation of the infection experience, supporting work motivation. These findings suggest that managers and colleagues recognize the importance of alleviating nurses' self‐blame, improving environments, and organizational support, enabling nurses to maintain achievement by reframing their experiences positively.

## Key Points

- This qualitative, inductive study explored the evolving feelings and perceptions of nurses who experienced occupational infection with COVID‐19 and their subsequent return to work during the late phase of the pandemic in Japan.
- Although negative emotions during infection may lead to dissatisfaction and a potential intent to leave the profession, support from managers and colleagues, along with personal reframing of experiences, can foster determination and motivation to return to work.
- Establishing supportive interactions that prevent nurses from feeling blamed or self‐reproached, improving workplace environments, and providing organizational efforts to nurture their growth and sense of accomplishment are essential. These findings offer important insights into the safe and continuous employment of healthcare workers during emerging infectious disease outbreaks.

## 1 Introduction

The first case of coronavirus disease 2019 (COVID‐19) was reported in Japan in January 2020. The infection spread rapidly, eventually leading to a global pandemic. In Japan, COVID‐19 was reclassified from a strictly managed category under the Infectious Diseases Control Law after 3 years, during which eight waves of infection occurred. According to the Ministry of Health, Labour and Welfare (2023), the total number of confirmed COVID‐19 cases from the first to the eighth wave (January 6, 2020, to March 2, 2023) was 33 168 104, with 72 009 reported deaths.

Preventing the spread of infection is extremely challenging, and numerous clusters have emerged in healthcare settings, resulting in widespread occupational infections among healthcare workers (Kameyama et al. 2023).

A systematic review by García‐Vivar et al. (2023) demonstrated that healthcare workers, including frontline nurses during the COVID‐19 pandemic, faced substantial psychological risks, with a high prevalence of insomnia (40.6%), depression (38.7%), post‐traumatic stress disorder (29.8%), and anxiety (29.5%). A qualitative review by Yao et al. (2023), examining the experiences and perspectives of nurses infected with COVID‐19, further highlighted the emotional burden and professional impact of infection, underscoring the need for strong support systems and effective coping mechanisms.

An online survey of 938 healthcare workers found that infected individuals had significantly higher risks of stress, anxiety, and depression than those not infected (Mohammadian Khonsari et al. 2021). A longitudinal study (2023) of 289 infected healthcare workers reported that 36.2% experienced persistent symptoms 6 months after infection, accompanied by marked declines in health‐related quality of life (HRQOL) (D'Ávila et al. 2023). These sustained physical and mental effects characterize COVID‐19. Evidence from earlier emerging infectious disease outbreaks, such as SARS, indicates that infected healthcare workers experience severe psychological distress, including fear of infection and isolation‐related loneliness, as well as social barriers, such as uncertainty and stigma that hinder return to work (Maunder et al. 2003). These postinfection psychosocial burdens likely impede workplace reintegration. In Japan, Ooshige and Ishitobi (2022) reported that a nurse infected with COVID‐19 experienced shock, guilt, and anxiety related to persistent symptoms. However, prior studies largely emphasize acute mental health effects during the early pandemic (Mohammadian Khonsari et al. 2021; Ooshige and Ishitobi 2022) or psychosocial barriers observed in earlier outbreaks (Maunder et al. 2003).

This study uniquely provides an in‐depth qualitative examination of the emotions and return‐to‐work experiences of occupationally infected nurses during the late phase of the pandemic, marked by its prolonged three‐year course and widespread access to vaccines and treatments. Understanding nurses' experiences at the time of infection and during workplace reintegration may inform long‐term support strategies, promote professional continuity, and help prevent occupational infections in future emerging infectious disease outbreaks.

## 2 Objective

This study aimed to elucidate the feelings experienced by nurses occupationally infected with COVID‐19 during the late phase of the pandemic in Japan, a period marked by a surge in cases from the time of infection to their return to work.

## 3 Methods

### 3.1 Study Design

This study employed a qualitative descriptive design.

### 3.2 Definitions of Terms

1. *Late phase of the COVID‐19 pandemic in Japan*

This term refers to the period between January 1 and September 30, 2022, corresponding to the sixth and seventh waves in Japan. This phase is characterized by an unprecedented surge in cases that place a significant burden on the healthcare system, despite occurring approximately 2 years after the initial outbreak when vaccines, treatments, and infection control measures have been established (Ministry of Health, Labour and Welfare 2023).

2 *Occupational infection*

This term refers to cases in which an individual tested positive for SARS‐CoV‐2 via polymerase chain reaction (PCR) or antigen testing during a workplace cluster. For the purposes of this study, it was not mandatory for the infection to have been officially diagnosed or declared occupational by the head of the affiliated institution (e.g., facility director or head of the infection control office).

3 *Feelings*

This term refers to the emotional reactions experienced by nurses following occupational exposure to COVID‐19 infection, extending beyond transient emotions. Feelings are defined as being closely linked to cognitive appraisals (thoughts/cognition) of events occurring within the organizational environment, such as infection and return to work, and are considered core elements of individuals' lived experiences (Elfenbein 2023). Specifically, it includes diverse subjective mental states experienced at the time of diagnosis, during recuperation, and after returning to work. Furthermore, feelings after returning to work are defined as past experiences that have been retrospectively recalled and assigned meaning through reflection over a period of approximately 5–6 months after the return.

### 3.3 Participants

Participants were selected using convenience and snowball sampling based on the following inclusion criteria:

1. Nurses employed at healthcare facilities between January 1, 2022, and September 30, 2022. Regarding the request for cooperation, invitation letters and informed consent forms were mailed to potential participants in advance, and interviews were conducted only after the signed consent forms had been returned.
2. Nurses who experienced an occupational COVID‐19 infection during their working hours.

Eleven nurses working in two healthcare facilities in the Kinki and Tokai regions of Japan participated in this study. Among them, nine were female (82%), and two were male (18%). Their years of nursing experience ranged from 2 to 33 years, and all were assigned to general wards. Ten participants received one to three doses of the COVID‐19 vaccine; however, the vaccination status of one participant could not be confirmed. All the participants experienced at least one symptom of COVID‐19, including fever, dyspnea, sore throat, nasal discharge, dysgeusia, anosmia, or fatigue. All 11 participants returned to work after the infection, although one was on leave at the time of the interview.

### 3.4 Data Collection

Data collection was conducted between August 2022 and February 2023, approximately 5–6 months after the participants experienced occupational infection. Semi‐structured, one‐on‐one interviews were conducted using an online videoconferencing system (Zoom). To ensure privacy and ethical standards, the researcher participated in a university laboratory, while the participants joined from private rooms at home or in their workplaces. A semi‐structured interview guide developed specifically for this study was used to explore the participants' emotions and experiences from the period before infection through their return to work.

### 3.5 Data Analysis

Data were analyzed using “Ueno's Qualitative Analysis Method” (Ueno et al. 2017), a type of qualitative thematic analysis. In the initial process, the verbatim transcripts created from the audio recordings were divided into sentence units (codes) to ensure that the original meaning was preserved. Next, these codes were analyzed inductively based on commonalities and similarities in meaning, increasing the level of abstraction to generate subcategories, categories, and core categories.

To ensure the validity and rigor of the analysis, the study was conducted by three authors, university faculty members specializing in psychiatric nursing, psychology, and infection control nursing, all of whom had experience in qualitative research. Discussions were repeatedly held until a consensus was reached.

### 3.6 Ethical Considerations

This study was approved by the Research Ethics Committee of the Graduate School of Nursing, Nagoya City University (Approval number: 22006). Participants were informed both orally and in writing about the purpose and outline of the study, the voluntary nature of participation, their right to withdraw at any time without disadvantage, the protection of personal information, and the possibility of the results being presented at academic conferences. Informed consent was obtained from all participants.

## 4 Results

Interviews were conducted once per participant, with an average duration of 41 min (range: 38–50 min). The analysis yielded six core categories, 14 categories, and 50 subcategories (Table 1). A large number of subcategories were deemed essential to accurately capture the emotional experiences of nurses, which were highly complex and multifaceted, spanning from the period before infection to their return to work. This approach facilitated a detailed depiction of the diverse feelings and experiences of individual nurses.

**TABLE 1:** Thoughts and feelings of infected nurses from diagnosis to return to work.

Core category	Category	Subcategory
Negative feelings upon contracting the infection	Shock of infection	It was painful and unexpected
		I wondered why I was the only one who got infected
		I was overwhelmed with anxiety
	Blamed by others	Feeling as though I was blamed for inadequate infection control measures
		Being concerned about colleagues' perceptions
		Wishing not to be blamed for contracting the infection
	Feelings of guilt and self‐blame	I felt sorry toward patients and colleagues
		I was afraid of transmitting the infection to my family or patients
Acceptance and composed feelings	Coming to terms with having been infected	I have come to terms with being infected
		I was not worried because my symptoms were mild
	Estimation of the route of infection	Estimated the likely route of infection
Dissatisfaction with the workplace environment and hesitation to return	Insufficient infection control measures at the hospital	The spread of the infection could have been prevented if the hospital had acted sooner
		PPE shortages after cluster outbreaks, anxiety
		Infection control education was ineffective
		They had followed the prescribed infection control measures
		A COVID‐19 response manual specific to general wards was needed
		There was no support from the hospital or public health center
		Desired more infection control training
	Concerns about physical condition upon returning to work	Being unsure if their symptoms would improve
		They felt anxious about whether it was truly appropriate to return to work
Appreciated support for returning to work	Gratitude for support during infection	The support from those around me during isolation was very helpful
		I did not feel anxious during isolation
		Colleagues at work did not blame me for being infected
		I did not have feelings of blaming anyone
	Managers' desire to protect infected nurses	I was worried about the nurses who were infected
		I made efforts to create an atmosphere without blame
Negative feelings after returning to work	Dissatisfaction while working after returning	It was physically and mentally tough immediately after returning
		I felt the allowances were unfair
		Anxiety about reinfection
		I wanted the busy ward to be understood
		I could not voice complaints about the hospital
		I was dissatisfied with insensitive comments from managers
		I feared being blamed if another cluster occurred
	Desire not to work at the current workplace	I want to work somewhere calm
		Reflecting uncertainty about continuing nursing to work as nurses
		Being away from work for a while
		Being unable to return immediately to their hometowns
Support for the motivation to continue working after returning	Determination to return to work	I am relieved that I have no after‐effects
		I decided to return for now
		I felt bad if I didn't return soon
		I want to continue nursing as before
		Returning was natural
	Gratitude toward workplace and colleagues	I hope measures will be taken going forward
		I was relieved that measures were taken after the cluster occurred
		Encouraging exchanges after returning
		I felt reassured by the department managing staff health
		I was grateful for the support and guidance after returning
	Transforming the experience of infection positively	To prevent recurrence, we have to be careful ourselves
		Their experience of infection would be useful for future nursing
		Reaffirm their nursing philosophy through reflection after infection

**FIGURE 1:**
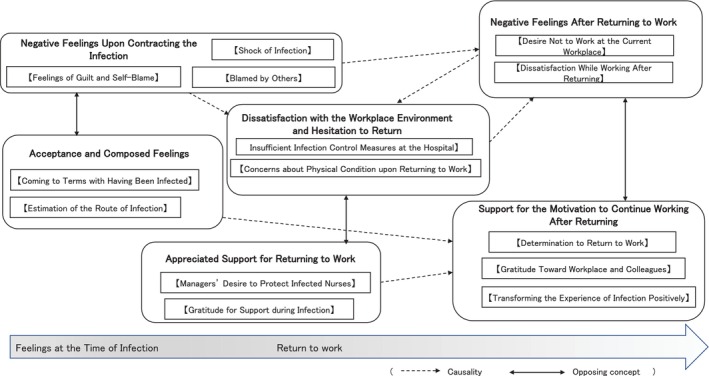
Concept map of thoughts and feelings from infection to postreturn to work.

Based on an examination of the relationships between these categories, a transition of feelings from the time of infection to returning to work was revealed (Figure 1).

### 4.1 Negative Feelings Upon Contracting the Infection

When nurses found themselves infected, a series of transitions occurred in the negative emotions they experienced upon the discovery of their infection. The process began with the shock of the infection, moved through concerns about criticism and responsibility from others, and eventually deepened into intense guilt and self‐reproaching behaviors.

Initially, the nurses expressed a sense of futility and felt that their long‐term efforts to restrict their activities outside the workplace and adhere to hospital infection control measures were in vain.

*I thought I would never get infected because I wore masks at the hospital. It was so painful to realize I got infected at the hospital, even though I had worked so hard for the past two years, not going anywhere and following all the restrictions*. (Nurse J)

This shock subsequently turned into a fear of criticism. Some nurses perceived repeated calls from management for strict infection control as a personal indictment of their inability to maintain perfect protocols amidst the overwhelming busyness of their daily duties.

*When management told us to be thorough with infection control, I felt like I was being blamed. With our daily work being busy, it was impossible to maintain perfect measures every time*. (Nurse I)

This sense of responsibility manifested as profound remorse and self‐blame for potentially becoming a source of infection and increasing the burden on colleagues remaining in the already strained wards.

*When I found out I was infected, many other nurses were also getting sick. I felt so sorry for the nurses I left behind in the ward*. (Nurse D)

### 4.2 Acceptance and Composed Feelings

After experiencing negative emotions, nurses, overwhelmed by self‐blame, sought to resolve their emotional confusion by reframing their infection as a reality. This core category manifested as an accepting reception of the infection and a rational estimation of the infection route.

Nurses came to understand “unpredictable risks”—such as patients who initially tested negative upon admission but later tested positive—not merely as clinical knowledge, but as a personal experience. They attempted to accept this situation as unavoidable.

*Patients undergo antigen testing before admission and enter only after a negative result is confirmed. However, since some patients turn positive after admission, I think it's inevitable that nurses might get infected by them*. (Nurse K)

After accepting the risk of infection, they calmly reflected on the route of infection and objectively evaluated their actions. They identified that close‐contact assistance to patients who are not wearing masks was a major contributing factor.

*[The infected patient] was a postoperative cardiovascular surgery patient experiencing delirium. They were struggling to breathe and couldn't wear masks. Even when I asked, they just couldn't do it … I wasn't able to implement [adequate] countermeasures there*. (Nurse B)

Nurses also reflected on their failure to take the best possible infection control measures amid their demanding workload.

*I realized I could have chosen to use an N95 mask, but I was so preoccupied with preoccupied with heavy workload that the idea of infection protection didn't even cross my mind. I regret that*. (Nurse D)

### 4.3 Dissatisfaction With the Workplace Environment and Hesitation to Return

The process of rationally estimating the infection route led to strong resentment toward what were perceived as “preventable circumstances,” resulting in dissatisfaction with the workplace and creating psychological barriers to returning to work. This core category manifested through criticism of the hospital's infection control measures as well as conflict and anxiety regarding physical health, which caused hesitation about returning.

Nurses pointed out delays in the hospital's initial response, noting that they became infected despite following infection control protocols.

*When the first positive case was identified, everyone (staff and other patients) tested negative via PCR, so the hospital did not stop new admissions or transfers. After that, positive cases appeared one after another. I felt that if different measures had been taken when the first case emerged, so many nurses wouldn't have had to test positive*. (Nurse A)

The depletion of supplies also intensified their psychological burden.

*I wish they had provided N95 masks the moment a positive patient was identified in the ward*. (Nurse G)

This dissatisfaction with the organization, combined with the profound anxiety experienced in the isolated environment during recuperation, transformed into psychological hesitation regarding their return to work.

*During my recuperation, I was isolated alone and anxious, wondering if my symptoms would get worse*. (Nurse K)

### 4.4 Appreciated Support for Returning to Work

Amid the isolation of quarantine and anxiety about returning, psychological support from colleagues and managers played a crucial role in physical and mental recovery and in alleviating the sense of isolation. This core category manifested as gratitude for the support received from those around them during the infection and the managers' desire to protect the infected nurses.

Connections with colleagues during isolation created a sense of solidarity beyond information sharing.

*I received an email from a colleague who had also been infected. Hearing that she had the same symptoms eased my anxiety a little, and it helped me get through the rest of my time. I felt that having someone follow up with you during recuperation is very reassuring*. (Nurse K)

For another nurse who expressed concern that “I was worried about whether the infection was spreading while I was away” (Nurse E), the timely sharing of information functioned as a form of psychological safety that helped maintain her motivation to return to work.

Specifically, managers' intentional considerations serve to break the chain of blame. Managers recognized both the burden on frontline staff and the guilt felt by those who were infected. One participant noted that her manager actively controlled the organizational tone by “telling everyone that we must not blame each other (whether infected or not)” (Nurse A).

### 4.5 Negative Feelings After Returning to Work

After returning to the frontline with insufficient physical recovery, nurses face structural workplace dissatisfaction and a lack of trust in the organization. These factors became serious triggers for them to reconsider career continuity. This core category manifested as persistent dissatisfaction after returning, physical and mental strain, and a growing desire to leave their current workplace.

*The ward remains strained even now, with bed capacity that cannot accommodate emergency patients. On top of that, we have an increasing number of patients with high care needs and urgency, but the number of nurses is decreasing. I am physically exhausted*. (Nurse D)

Furthermore, nurses felt poignant resentment toward instructions from the management that were not suitable for the reality of the clinical setting.

*There was a mismatch between what management said and the actual situation on the ground (…). I felt that they should look at frontline reality before giving orders*. (Nurse I)

One nurse, reflecting on prolonged symptoms, such as a chronic cough, stated:

*I probably had the most persistent cough in the ward. It made me feel like I should take better care of myself. I started thinking that it might be okay to prioritize my work‐life balance*. (Nurse C)

Similarly, another nurse reached her physical and mental breaking point:

*I was not in my best form at all. My cough lasted so long that I was anxious about my ability to work. Around the second or third day after returning to work, I felt closest to wanting to quit my job*. (Nurse H)

Under these circumstances, some were “currently on a leave of absence” (Nurse C), highlighting the profound and long‐lasting impact of infection on mental health and professional continuity.

### 4.6 Support for the Motivation to Continue Working After Returning

Despite physical and mental exhaustion following infection and dissatisfaction with the workplace, nurses maintained motivation through improvements in the postreturn work environment and support and mutual encouragement from managers and colleagues. This motivation consisted of three aspects: determination to return to work, gratitude toward the workplace and colleagues, and transformation of the infection experience into a positive asset.

*I love the nursing profession. It's the path I chose because I was interested in it, so I don't feel like quitting*. (Nurse E)

The nurses also demonstrated a proactive attitude, seeking to link their experiences with personal growth by acquiring new knowledge and perspectives on infection control and patient care.

*By experiencing the infection myself, I've become able to provide a new kind of nursing care. Right now, the desire to continue to work as a nurse is stronger*. (Nurse H)

*I feel more committed to following infection prevention measures more strictly than I was before the cluster occurred*. (Nurse G)

Furthermore, the measures and guidance implemented in the workplace after their return restored trust and served as a foundation for the motivation to work.

*The infection control team visits us constantly and provides guidance. When the supplies run low, they gather them for us. Being able to ask questions over the phone whenever I am unsure is a great help*. (Nurse J)

Finally, emotional solidarity and words of encouragement exchanged with colleagues and managers supported the nurses in harsh clinical settings and provided a profound sense of security.

*We told each other, 'This will end eventually; it won't last forever. Let's all do our best together to prevent it from spreading further*. (Nurse K)

*The nurse manager and assistant manager in my ward also contracted COVID‐19. They repeatedly told me, 'It's not just you, so please don't carry the burden of responsibility alone.' I was so grateful for that*. (Nurse F)

## 5 Discussion

### 5.1 Feelings of Guilt and Frustration Experienced by Nurses Infected With COVID‐19

Analysis of the experiences of nurses who contracted COVID‐19 occupationally during the late phase of the pandemic, revealed intense feelings of guilt and self‐reproach toward others, often exceeding the shock of the infection itself. This phase was marked by the rapid spread of emerging variants, a sharp increase in cases (Ministry of Health, Labour and Welfare 2023), and large‐scale infection clusters (Sato et al. 2023). Under these circumstances, concern about the potential impact of their infection on colleagues and patients likely intensified nurses' feelings of guilt and self‐reproach. This finding aligns with prior qualitative studies of infected nurses (Ooshige and Ishitobi 2022; Shinkai et al. 2022ab). Ooshige and Ishitobi (2022) reported guilt related to no longer working alongside colleagues, while we found that nurses felt apologetic about becoming infected and reproached themselves for patient deaths (Shinkai et al. 2022a, 2022b). These responses may reflect the strong professional pride and sense of responsibility inherent in nursing (Japanese Nursing Association 2021). However, guilt has been shown to trigger acute stress reactions and severe psychological trauma among healthcare workers (Cavalera 2020), potentially hindering return to work and contributing to turnover (Falatah 2021). Workforce shortages, in turn, compromise care quality, increase workload stress, and elevate risks of burnout (Galanis et al. 2021) and depression (Sato et al. 2023). These findings underscore the importance of organizational support to mitigate guilt among infected nurses. In addition to guilt, nurses reported helplessness and frustration when unable to implement adequate infection control measures for patients with delirium or high fever who struggled to wear masks.

Frustration was further exacerbated by insensitive remarks from managers, which nurses perceived as reflecting a lack of understanding of ward‐level workload after their return. This disconnect may have contributed to thoughts of leaving their workplace.

Support from facility administrators, particularly recognition and appreciation, has been identified as essential for healthcare workers caring for patients with COVID‐19 (Pellikka et al. 2024). Positive acknowledgment from management is associated with improved job engagement and well‐being (Oyama et al. 2023). Given the chronic stress faced by frontline nurses during the pandemic, consistent encouragement and recognition of their efforts to prevent infection clusters remain critically important.

### 5.2 Changes in the Feelings of Infected Nurses According to the Timing of the Outbreak

A study conducted by us during the early stages of a domestic outbreak revealed intense shock, anxiety, and confusion associated with an unknown infectious disease in clinical settings (Shinkai et al. 2022a). In contrast, the participants in this study were infected during the late phase of the pandemic—approximately 2 years after the initial outbreak, when vaccinations had become widespread, the characteristics of the pathogen had been clarified, and prevention protocols were well established. Consequently, while “shock” remains present in their narratives, their cognitive appraisal had shifted from the fundamental fear seen in the early stages to an “acceptance of professional risk.” This disparity suggests that advancements in knowledge and response strategies regarding infectious diseases influenced nurses' psychological preparedness.

The importance of “creating a workplace free from blame” and “solidarity among colleagues” identified in this study provides universal insights into sustaining professional continuity during any nosocomial infection, regardless of the epidemic phase or the type of pathogen.

However, what distinguishes this experience from conventional nosocomial infections is the prolonged nature of the pandemic, spanning over 3 years, and the persistent physical and mental exhaustion that follows the infection. As suggested by cases involving leaves of absence, symptoms such as lingering fatigue and chronic coughing do not merely end after a standard recovery period; they also encompass the risk of Long COVID. This represents the unique impact of COVID‐19 not typically observed in standard hospital‐acquired infections.

These findings suggest the necessity for comprehensive long‐term support, including mental health care, that addresses the long‐term career risks emerging under the unique circumstances of a pandemic.

### 5.3 Supports for Continued Work Motivation After Returning and Implications for Nursing

One of the factors that led to their determination to return to work amidst the emergence of the cluster outbreak was rooted in their sense of gratitude toward their workplace and colleagues. A review by Kisely et al. (2020), which examined strategies for maintaining and promoting the mental health of healthcare workers during outbreaks of emerging infectious diseases, including COVID‐19, identified the presence of supportive colleagues, a sense of support, and trust in organizational infection control measures as protective factors for mental health. Similarly, in this study, although nurses initially hoped “I didn't want to be blamed for being infected” upon learning of their infection, they developed a deep sense of gratitude for support during infection through the assistance provided during their isolation period. They expressed appreciation for “I was not criticized by my colleagues for being infected,” and for improvements in the workplace after returning, as well as for the mutual encouragement shared with supervisors and coworkers. These findings underscore the importance of fostering workplace environments that promote motivation, teamwork, and mutual recognition.

The second factor was the ability to positively transform the experience of infection. Upon diagnosis, the nurses experienced emotional distress and shock from being infected in the ward, along with feelings of frustration and disappointment due to a lack of acknowledgment from their managers. However, with the support of their supervisors and staff, they developed a sense of gratitude. As they reflected on their growth as nurses and reaffirmed their perspectives on nursing, as exemplified by comments such as “I feel I've gained more skills,” they found a sense of accomplishment and professional fulfillment.

As the spread of COVID‐19 persists in Japan, this study highlights the importance of continuous institutional support in sustaining nurses' motivation to work. Specifically, encouragement and recognition from managers and colleagues are vital for preventing self‐blame and feelings of criticism. Furthermore, the findings emphasize the need for workplace improvements and opportunities for nurses to continue building experience and a sense of professional accomplishment.

## 6 Limitations and Future Challenges

This study used data from only two facilities, thus limiting the generalizability of the findings. Additionally, the small sample size and the timing of the survey, conducted 5 months after infection, pose limitations, as the subsequent development of long COVID symptoms and the continued progression of the pandemic may have influenced the participants' mental health.

Future challenges include developing a quantitative questionnaire based on the findings of this study and conducting large‐scale surveys. This approach provides broader insights into the experiences of occupationally infected healthcare workers and informs the development of effective support strategies.

## 7 Conclusion

Nurses who were occupationally infected with COVID‐19 during the late phase of the pandemic experienced shock upon infection, guilt and self‐blame toward others, and a sense of being blamed for inadequate infection control measures. They perceived that insufficient infection control measures at the hospital contributed to the spread of infection. Persistent dissatisfaction and hesitation before returning to work were suggested as factors leading to postreturn dissatisfaction and intention to leave the job. Despite the challenges of returning, the experience of infection fostered a renewed motivation to proactively prevent future infections. Encouragement and support from managers and staff, improvements in the workplace environment, the accumulation of nursing experience, and a sense of accomplishment were identified as key factors for sustaining motivation to continue working after returning.

## 8 Relevance for Clinical Practice

The findings provide crucial insights into specific support measures for nurses during future emerging infectious disease outbreaks and how healthcare institutions should prepare for similar crises. Hospitals can contribute to nurses' safe continued employment and the prevention of occupational infections by building a supportive work environment, ensuring the availability of the necessary equipment and facilities for occupational infection prevention, and offering continuous support that allows their experiences to foster growth.

## Author Contributions

**Noriko Shinkai:** conceptualization, investigation, funding acquisition, writing – original draft, writing – review and editing, methodology, project administration, data curation, supervision. **Kayoko Ohnishi:** conceptualization, methodology, validation, writing – review and editing, data curation. **Hisako Yano:** conceptualization, methodology, validation, writing – review and editing, data curation.

## Funding

This work was supported by Japan Society for the Promotion of Science (JSPS) KAKENHI (22K17441).

## Ethics Statement

This study was approved by the Research Ethics Committee of the Graduate School of Nursing, Nagoya City University (Approval No. 22006).

## Conflicts of Interest

The authors declare no conflicts of interest.

## Supporting information


**Data S1:** SRQR Checklist.

## Data Availability

The data generated in this study are not publicly available due to the privacy and personal information protection of the participants.
